# Managing Female Athlete Health: Auditing the Representation of Female versus Male Participants among Research in Supplements to Manage Diagnosed Micronutrient Issues

**DOI:** 10.3390/nu14163372

**Published:** 2022-08-17

**Authors:** Ella S. Smith, Alannah K. A. McKay, Megan Kuikman, Kathryn E. Ackerman, Rachel Harris, Kirsty J. Elliott-Sale, Trent Stellingwerff, Louise M. Burke

**Affiliations:** 1 Mary MacKillop Institute for Health Research, Australian Catholic University, Melbourne, VIC 3000, Australia; 2 Wu Tsai Female Athlete Program, Boston Children’s Hospital and Harvard Medical School, Boston, MA 02115, USA; 3 Female Athlete Performance and Health Initiative, Australian Institute of Sport, Canberra, ACT 2617, Australia; 4 Perth Orthopaedic and Sports Medicine Research Institute, West Perth, WA 6005, Australia; 5 Institute of Sport, Manchester Metropolitan University, Manchester M15 6BH, UK; 6 Canadian Sport Institute-Pacific, Institute for Sport Excellence, 4371 Interurban Road, Victoria, BC V9E 2C5, Canada; 7 Exercise Science, Physical and Health Education, University of Victoria, Victoria, BC V8P 5C2, Canada

**Keywords:** women, physical activity, menstrual status, oral contraceptive, nutrition, nutrient deficiency, calcium, iron, vitamin D

## Abstract

Micronutrient deficiencies and sub-optimal intakes among female athletes are a concern and are commonly prevented or treated with medical supplements. However, it is unclear how well women have been considered in the research underpinning current supplementation practices. We conducted an audit of the literature supporting the use of calcium, iron, and vitamin D. Of the 299 studies, including 25,171 participants, the majority (71%) of participants were women. Studies with exclusively female cohorts (37%) were also more prevalent than those examining males in isolation (31%). However, study designs considering divergent responses between sexes were sparse, accounting for 7% of the literature. Moreover, despite the abundance of female participants, the quality and quantity of the literature specific to female athletes was poor. Just 32% of studies including women defined menstrual status, while none implemented best-practice methodologies regarding ovarian hormonal control. Additionally, only 10% of studies included highly trained female athletes. Investigations of calcium supplementation were particularly lacking, with just two studies conducted in highly trained women. New research should focus on high-quality investigations specific to female athletes, alongside evaluating sex-based differences in the response to calcium, iron, and vitamin D, thus ensuring the specific needs of women have been considered in current protocols involving medical supplements.

## 1. Introduction

Athletes commonly use medical supplements, defined as “supplements used to prevent or treat clinical issues including diagnosed micronutrient deficiencies” [1,2,3]. Indeed, 4–87% report the use of a vitamin and/or mineral supplement [2], with some evidence for higher usage rates among female athletes [4]. Although micronutrient deficiencies are common in the community [5], athletes may have elevated risks due to increased nutrient turnover [3] and/or sub-optimal vitamin/mineral consumption [6]. Since micronutrients play central roles in numerous bodily functions, including energy production, gene transcription, bone health, immunity, and inflammatory responses [3,7,8,9,10], deficiencies can result in elevated rates of illness and injury, reduced training responses, and impaired athletic performance [3,11,12,13].

Medical supplements should be used under the supervision of an accredited dietician and/or medical practitioner [8] involving evidence-based protocols. However, recent scrutiny of the sports nutrition literature suggests that female athletes are not adequately considered in the supporting research [14]. We employed our audit protocol [15] to evaluate the representation of high-performance female athletes in the science informing the use of three medical supplements (iron, calcium, and vitamin D) commonly used to treat/prevent nutrient deficiencies. Our aim was to examine the quality and quantity of research underpinning the use of these supplements in typical sports nutrition/medicine practice.

## 2. Materials and Methods

This audit was conducted according to the methods comprehensively outlined by Smith et al. [15]. Although gender and sex are separate constructs, in this manuscript, “woman” and “female” are used interchangeably because the audited studies did not specify verification of sex or gender identity.

### 2.1. Search Strategy

An electronic literature search of PubMed was conducted using the following terms: “(athlete OR sport OR healthy) AND (**supplement specific terms**) AND (exercise OR performance OR endurance OR aerobic OR strength OR power OR anaerobic OR adapt* OR treatment) NOT animal NOT rodent NOT diabetes NOT obese NOT overweight NOT disease NOT bacteria NOT patient NOT illness NOT in vitro NOT hospital NOT children NOT elderly NOT infection”. Supplement-specific terms were: (1) calcium OR CA2+; (2) iron OR hepcidin OR anaemi* OR anemi* OR iron deficien* OR heme OR haem; and (3) vitamin D. The following additional search terms were utilised for calcium: “NOT Coronary artery calcium NOT Coronary artery disease NOT Calcium oxalate”. Searches were exclusive to original papers with human participants, published in English, without date restrictions and current to 1 February 2022. Selected review articles for each supplement were screened to identify further relevant papers not detected in the primary search.

### 2.2. Data Extraction

**Figure 1:**
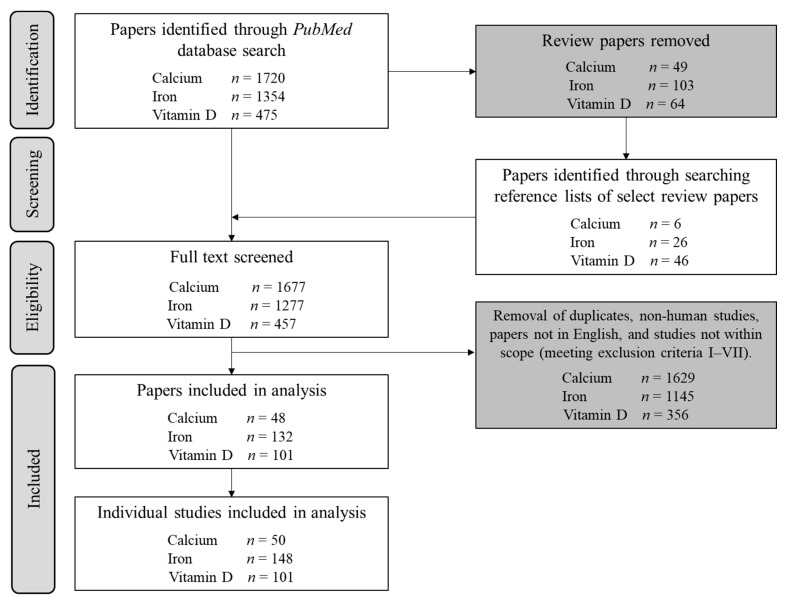
Flowchart illustrating the initial screening procedure for each supplement audited, alongside the total number of individual studies included for extraction of metrics A–F.

Initial screening was conducted on Rayyan software [16] (Figure 1). Duplicates and review papers were removed, alongside papers meeting the exclusion criteria: (i) untrained participants >50 years (i.e., older populations other than identified masters athletes), (ii) children (cut-off ages determined by individual sports), (iii) presence of lifestyle diseases (e.g., hypertension) or smoking, (iv) multi-ingredient products, unless the supplement of interest was the primary ingredient, (v) failure to investigate the supplement as primary outcome/independent variable, (vi) outcomes irrelevant to performance, health, or indirect associations with performance/health, and (vii) failure to explicitly state the sex, or the ratio of male: female participants. Each dataset in papers involving multiple studies was analysed individually. Hereafter, the term “paper” refers to the entire publication, whereas “study” describes discrete investigations within a paper. Eleven studies in the search results, which investigated calcium and vitamin D combinations without either supplement in isolation, were deemed not to meet inclusion criteria. Multiple studies which utilised calcium as a placebo condition, rather than an independent variable, were also excluded from analysis.

After the primary screening, the following metrics were extracted, as described elsewhere [15]: (A) study population; (B) athletic/fitness calibre [17]; (C) menstrual status (methodological consideration graded as bronze/silver/gold standard or ungraded); (D) research theme: performance/health/indirect associations with performance or health; (E) journal publication dates and study impact factor (IF) correct as of 1 March 2022; and (F) sample size. Participants of “tier 0” athletic calibre [17] (sedentary; metric B) were included due to the relevance of mechanistic studies not requiring an exercise condition. Studies of medical supplement use among pregnant women were classified separately for menstrual status (metric C) without being graded. Regarding metric D, in scenarios where studies measured both “health” and “performance” outcomes, a “performance” classification was prioritised to capture studies investigating performance outcomes from correcting a nutrient deficiency. Studies examining multiple individual supplements (e.g., calcium and iron administered separately within a single study) were included separately for each supplement. We also quantified study aims, such as treating a clinical deficiency, supplement bioavailability/absorption, different supplement formulations, clinical outcomes, or the use of a food-first/food fortification approach. Where possible, we quantified the dose of each supplement (both amount and timing) and modality of ingestion (e.g., oral or intravenous).

### 2.3. Statistical Analysis

Statistical analyses were performed in R Studio (v3.5.2) with statistical significance accepted at *p* ≤ 0.05. Frequency-based metrics (A–D) were reported as percentages of the total studies/participants, with ranges provided for outcomes across supplements or populations. Except for calcium, inspection of the histograms for journal IF, Altmetric scores, and male/female specific sample size revealed non-normal data, so all results (metrics E and F) were reported as median ± interquartile range (IQR). Metrics E and F were analysed with a generalised linear mixed model to account for unequal group sizes, using supplement and population as fixed effects. However, implementing AIC stepwise regression demonstrated that no additional variance was accounted for by these predictors, and it was evident that these variables failed to explain the dependent variable (likely due to highly skewed and missing data). Consequently, this analysis is not presented; however, it can be noted that no significant differences between population or supplement were noted within any model. The proportion of studies achieving an Altmetric score > 20 was assessed in a binary manner to demarcate studies receiving greater attention than others [18].

## 3. Results

**Figure 2:**
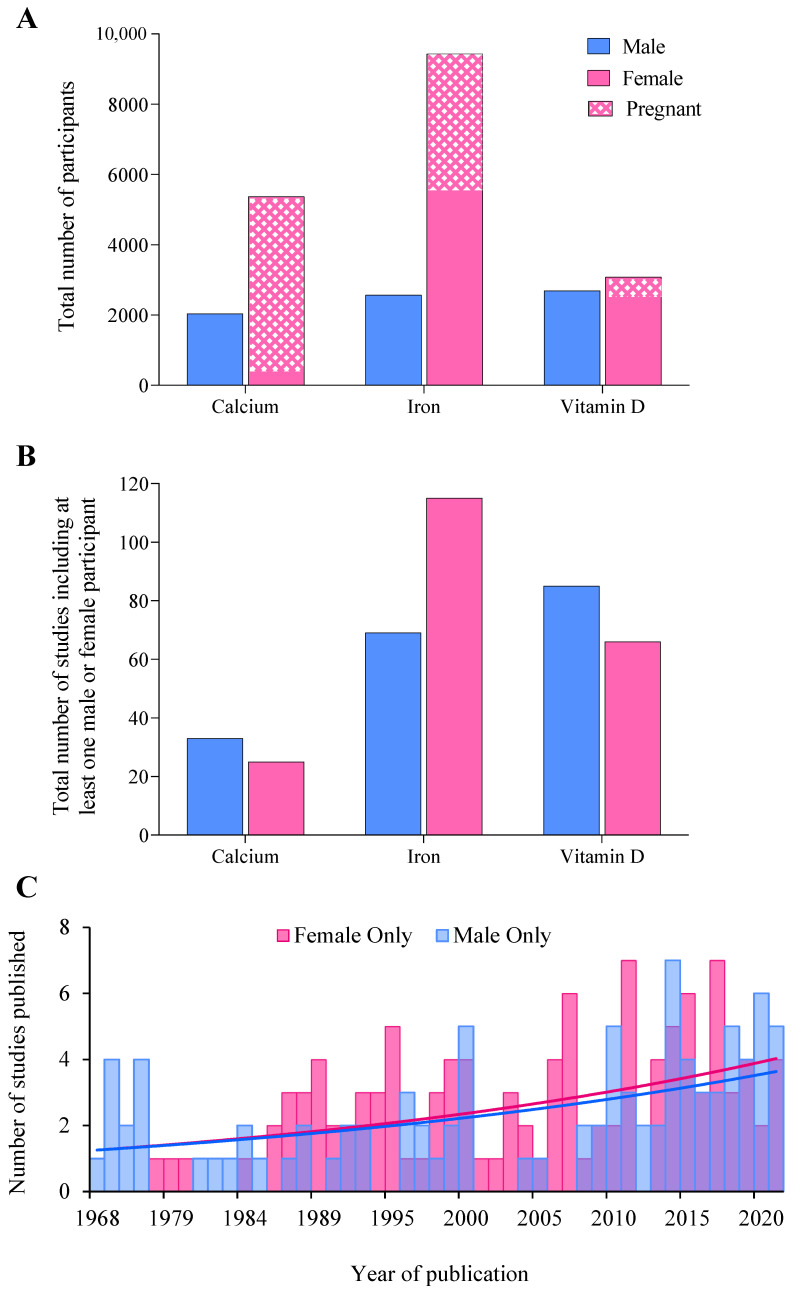
(**A**) The total number of male, female, and pregnant participants included across each supplement audited, (**B**) the total number of studies including at least one male or female participant, and (**C**) histogram displaying the total number of studies published in an exclusively male or female cohort between 1968 and 2021 across all supplements.

Across all supplements, 299 studies involving 25,171 participants met inclusion criteria (Figure 1). The majority (71%, 17,895 individuals) of participants were women (Figure 2A), while 69% of studies included at least 1 woman (Figure 2B). Male and female participation were comparable in studies of vitamin D supplementation (2668 men and 3086 women), whereas rates of female participation were 2.6 and 3.7 times higher than for males in calcium and iron studies (Figure 2A). Comprehensive results for each supplement are detailed in the Supplementary Material (Figures S1–S3). The annual publication rate of studies in exclusively female populations was similar to publications of male-only cohorts, at a mean of three studies per year. Moreover, the trajectory of publication rate was nearly identical between sexes, increasing from an average of ~one study per year in 1968 to ~four studies in 2021 (Figure 2C).

### 3.1. Population and Sample Size

**Figure 3:**
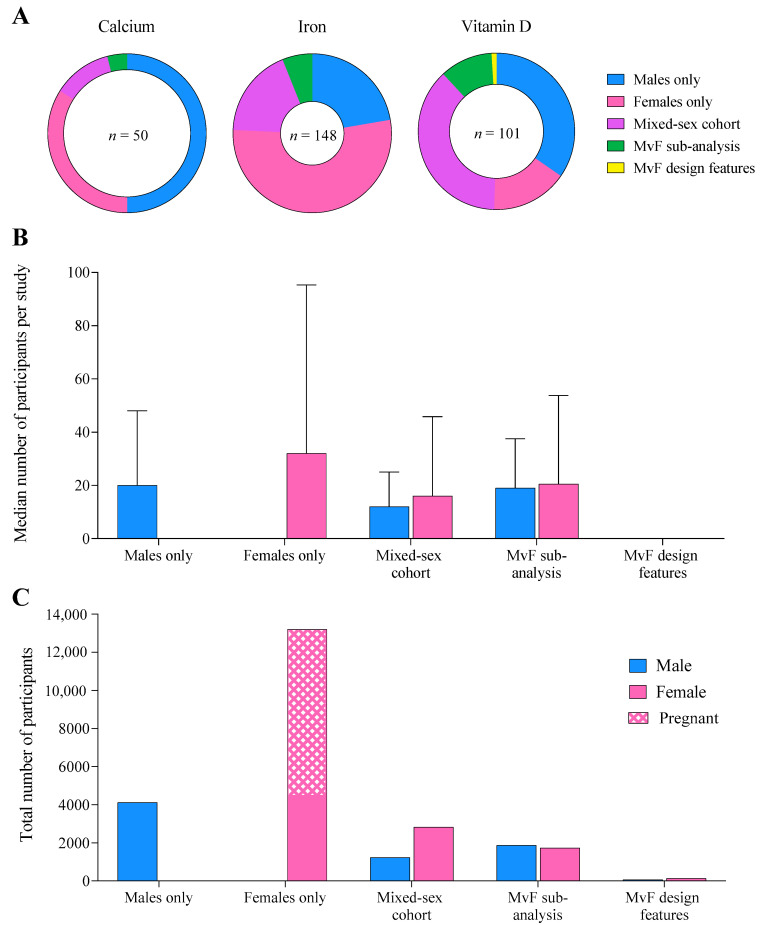
(**A**) The proportion of studies across each supplement published in each population, (**B**) the median number of male and female participants per study, and (**C**) total number of male, female, and pregnant participants combined across all supplements, according to the population studied.

Across all supplements, 16–53% of studies investigated female-only cohorts, similar to the 22–50% investigating exclusively males (Figure 3A). Mixed-sex cohorts were less prevalent, accounting for 12–37% of studies, while 4–11% utilised an MvFsub design, and one study employed a methodological design specifically comparing sex-based responses (MvFdes) to vitamin D supplementation. Across all supplements, 26 studies totalling 8909 participants involved pregnant women.

Median sample size was largest and most variable in female-only cohorts (32 ± 63), compared to male-only (20 ± 28), mixed-sex cohort (12 ± 13), and MvFsub cohorts (19 ± 19, Figure 3B). Women generally accounted for a larger proportion of the cohort than men in studies investigating both sexes. Female-only study designs included a total of ~3 times more participants than male-only cohorts (13,203 women vs. 4110 men, Figure 3C), while substantially fewer individuals (2006 men and 2010 women) participated in studies evaluating sex differences in supplement responses (MvFsub and MvFdes).

### 3.2. Athletic Calibre

**Figure 4:**
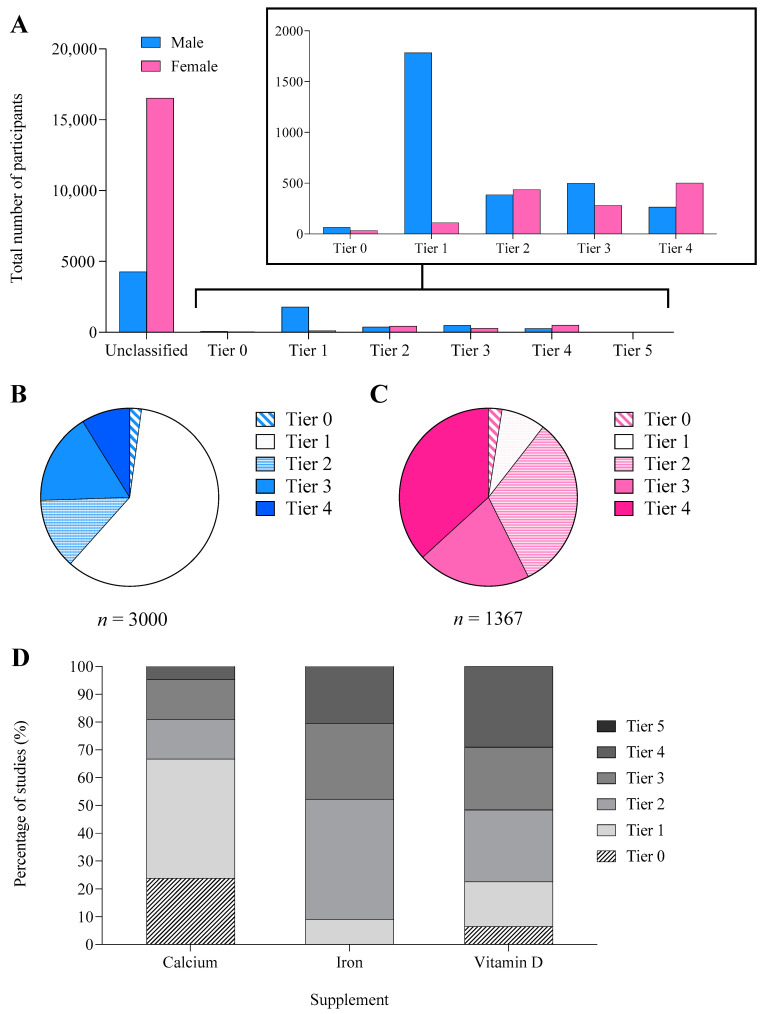
(**A**) Total number of male and female participants and the proportion of male (**B**) and female (**C**) participants in each athletic tier [19]: Tier 0 (sedentary), Tier 1 (recreationally active), Tier 2 (trained/developmental), Tier 3 (highly trained/national), Tier 4 (elite/international), and Tier 5 (world-class). (**D**) The proportion of participants in each athletic tier separated by supplement. Only classifiable participants are reported in all figures.

Although 68% of studies did not provide adequate information to classify participants’ athletic/fitness calibre, the number of classifiable men was ~2.2 times greater than the number of women. Most women were categorised as Tier 4 (37%) or Tier 2 (32%), whereas most men were Tier 1 (60%, Figure 4A). Classified female participants were typically of a higher calibre (57% from Tiers 3–4, 37% from Tier 4) than male participants (26% from Tiers 3–4, 9% Tier 4, Figure 4B,C).

### 3.3. Menstrual Status

**Figure 5:**
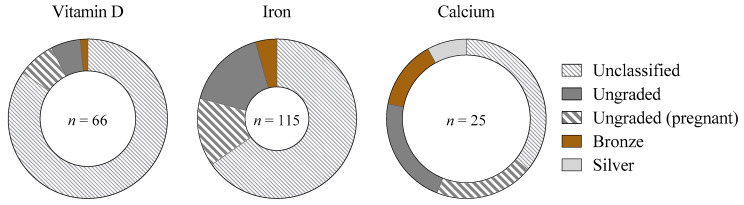
The proportion of studies classified into each tier [15] according to the standard of methodological control concerning ovarian hormonal profiles, as a proportion of the total number of studies including women across each supplement. Gold-standard (best-practice methodologies as detailed by Elliott-Sale et al. [19]), silver/bronze (achieve some, but not all, methodological considerations), ungraded (menstrual status is defined, but methodological control is insufficient to award bronze/silver/gold) or unclassified (insufficient information to provide a distinct participant classification, or a mixed female cohort in which individual menstrual status cannot be differentiated). Studies including pregnant women are highlighted separately to distinguish from studies not classifying participants.

A total of 66 (32%) of the 206 studies including women defined menstrual status, according to the following classifications: naturally menstruating (23 studies), HC users (4 studies), pregnant women (26 studies), and mixed, but identifiable (13 studies). The overall standard of methodological classification was poor: of 40 studies defining participant menstrual status (excluding pregnancy), 28.5 (71%) were ungradable, 9.5 (24%) were bronze-standard, and 2 (5%) were silver [15]. No studies achieved gold-standard [15] classification (Figure 5). Studies on naturally menstruating women (n = 23) and mixed-sex cohorts including naturally menstruating women (n = 13) achieved a mean of 0.4 out of the 5 criteria justifying a eumenorrheic classification [19], with a high score of 4 achieved by 1 mixed-sex study of calcium supplementation [20].

### 3.4. Performance vs. Health Research Themes

**Figure 6:**
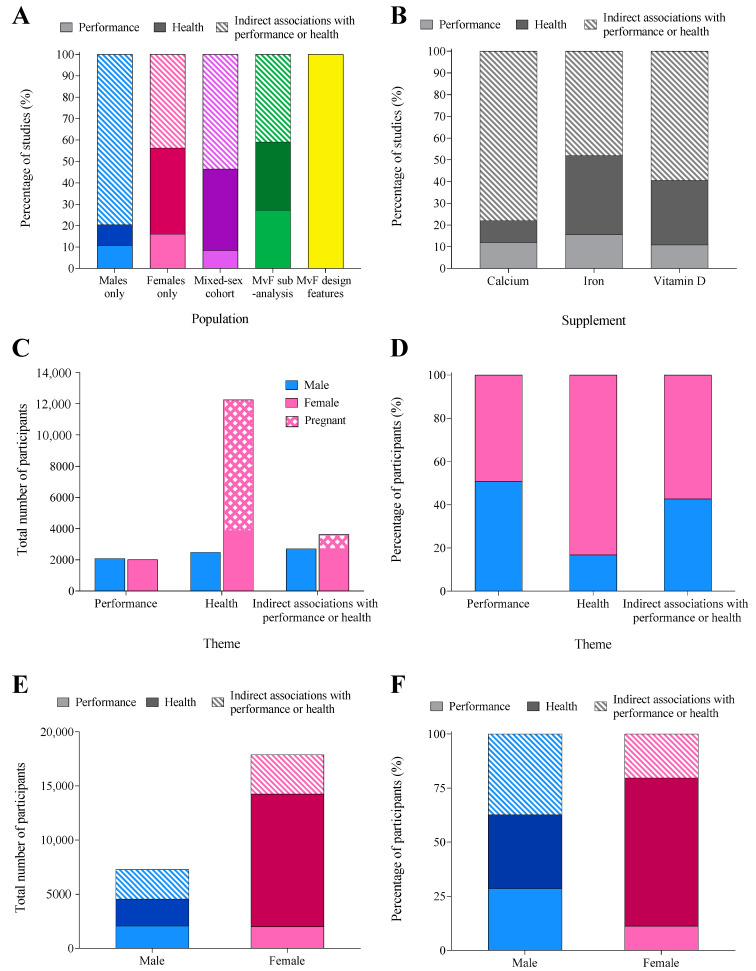
The percentage of studies in each theme: performance (direct performance outcomes), health (outcomes related to health status/condition), and indirect associations with performance/health (physiological or psychological adaptation/response that may subsequently transfer to athletic performance/health), separated by (**A**) population and (**B**) supplement. The total number (**C**) and proportion (**D**) of male and female participants in each research theme. The total (**E**) and proportion (**F**) of male and female participants involved in studies measuring outcomes of performance, health, or indirect associations with performance or health.

Across all supplements, indirect markers of performance/health were the most frequently studied theme (57% of studies), followed by health outcomes (30% of studies), and then performance measures (13%). This pattern was mirrored across individual populations and supplements (Figure 6A,B). Studies measuring health outcomes employed the largest participant number (14,734 individuals, including 83% (12,252) women, Figure 6C). Women out-represented men in studies of indirect associations with performance/health (57%) and health (83%); however, males and females were equally represented (~50%) in performance outcomes (Figure 6D). Male participants were relatively evenly split across all themes, while studies including female participants were dominated by health outcomes (Figure 6E,F).

### 3.5. Journal and Study Impact

**Figure 7:**
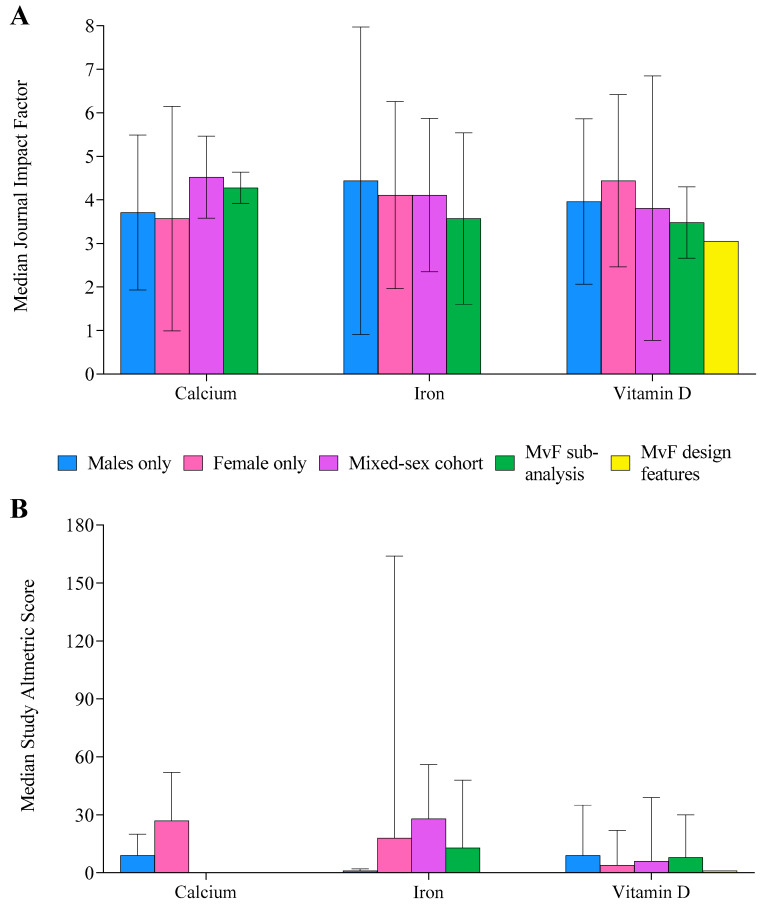
Median journal impact factor (**A**) and Altmetric score (**B**) for each supplement, separated by population studied. Male (M), female (F). Error bars display the interquartile range.

The median IF of journals in which studies were published was 3.9 ± 2.3 (Figure 7). Altmetric scores were only available for 45% of studies (n = 133) and were highly variable, with a median score of 7 ± 28 (Figure 7). Overall, 16% of studies (n = 47) achieved Altmetric scores > 20. Although studies investigating MvFsub had the greatest proportion of scores > 20 (n = 5 studies, 23%), the total number of such studies (n = 22) was low. Overall, vitamin D (n = 24 studies, 24%) and iron (n = 20 studies, 14%) had more studies with Altmetric scores > 20 than the calcium literature (n = 3, 6%).

### 3.6. Supplement-Specific

The literature base for each supplement included 50 studies of calcium, 101 studies of vitamin D, and 148 studies of iron supplementation. Across supplements, 29% of studies investigated strategies to treat micronutrient deficiencies, 18% examined physiological responses/adaptations, 15% investigated the efficacy of different formulations or dosing protocols, and 8% measured absorption/bioavailability. Supplement dosage quantity, modality, and frequency varied across supplements (Table 1).

### 3.7. Athlete-Specific Literature

Across the 32% (96/299) of studies that could be classified into an athletic tier, 41 were considered Tiers 3–4 [17] (i.e., at least highly trained/national level), of which 29 included women. When separated by supplement, Tier 3–4 athletes participated more frequently in studies of iron (48% of participants, *n* = 21 studies) and vitamin D supplementation (52%, *n* = 16 studies), while calcium (19%, *n* = 4 studies) involved the lowest percentage of athletes from these tiers (Figure 4D). Of the 29 studies including female athletes (Tiers 3–4), menstrual status was unclassified in 22, and 2.3 of the remaining 7 studies achieved a bronze standard of methodological control with the remainder ungraded. Outcome measures were evenly split among studies including Tier 3–4 athletes, with 36% examining indirect associations with performance/health or direct health measures and 27% investigating performance. Most (44%) studies investigating athletes examined strategies to treat a nutrient deficiency.

## 4. Discussion

We undertook a literature audit to examine the representation of women in the evidence base guiding the use of medical supplements (calcium, iron, and vitamin D) to prevent/treat clinical issues in athletes [1,2,3]. Although we were interested in the use of these supplements in sports nutrition, we audited the broader literature that typically informs practices around medical supplement use. Women accounted for the majority (71%) of participants, while 69% of studies included at least one woman. Female-only study designs included ~3 times more participants than studies investigating exclusively males, mostly due to the large number of studies involving pregnant women. Indeed, 26 studies investigating pregnant women accounted for 9% of the total studies but contributed 50% (8909 individuals) of the total female participants. Female representation in this medical supplement field was greater than that previously observed across other themes [14,21,22,23,24], which may reflect a higher prevalence of micronutrient deficiencies or sub-optimal intake, particularly iron deficiency/anaemia, among women [25,26]. However, there was minimal exploration of sex-specific differences in supplement response, with just 4–11% of studies allowing comparisons between men and women in the statistical analysis and only one study designed to investigate sex-based responses. Furthermore, widespread inadequacies in methodological design regarding menstrual function preclude insights into the effects of these parameters on micronutrient metabolism and supplement responses, an important area for athletes because of the potential for acute interactions with strenuous exercise [27,28,29,30,31,32].

Although we predetermined that our audit would include studies involving non-active participants because of the relevance of general research on medical supplements, we were also interested in identifying the calibre of athletes involved. Here, studies involving men were twice as likely to provide information on participant training status/calibre. This reflects the large number of studies in females in which pregnancy apparently negated any interest in the sporting involvement of the nearly 9000 participants. Of the females who were classified, 57% (785/1367 individuals, representing 13% of all female participants) were defined as Tier 3–4 athletes [17], while most (60%) classified men were Tier 1 (1784/3000 individuals, 25% of male participants) with 26% from Tiers 3–4 (*n* = 766 individuals). The apparently high involvement of Tier 3–4 athletes is inflated by the large number of investigations (*n* = 208, 68%) where participant classification was not possible. Importantly, there were no studies investigating world-class athletes [17] across any supplement.

Despite the high number of studies in women, the overall quality of menstrual status classification and methodological control of ovarian hormones was poor, mirroring our previous findings among performance supplements [14] and acute carbohydrate ingestion [33]. Overall, 32% of all studies including women defined participant menstrual status, but none achieved the gold standard of methodological control [19]. Moreover, studies among athletic (*n* = 29, Tiers 3–4) females achieved only bronze status [15] at best (8%), and none investigated HC users or women with menstrual irregularities, despite the prevalence of these populations among female athletes [34,35,36,37]. This inadequate classification was compounded by some confusing language, with some studies stating participants had a “regular menstrual cycle” but also describing HC use in the same population, despite these scenarios being mutually exclusive.

Indirect markers of performance/health (e.g., changes in micronutrient status, supplement absorption/bioavailability, supplement formulations/protocols) were the most frequently investigated outcome across all populations and supplements. Studies including female participants were dominated by health measures (e.g., treating micronutrient deficiencies, or outcomes related to injury/illness); removing the 26 studies including 8909 pregnant participants reduced the percentage of women involved in studies of health outcomes from 68% to 44%. Investigation of the effect of medical supplements on athletic performance is sparse; a performance outcome was included in only 40 of 299 studies. Furthermore, since only 11 of these 40 studies (9 of which included women) included Tier 3–4 participants [17] the application of the results is limited.

Concerningly, our audit located (but excluded) 22 studies that failed to identify participant sex, reinforcing an indifference to the existence/importance of sex differences. The mean publication year for these 22 studies was 2012, with 15 studies published in the last 10 years. This ignores growing awareness of the under-representation of women in sport science/sports medicine research [15,21,22,24] and increased interest in sex-specific responses to interventions [22,23,38]. It appears that even in research areas in which outcomes may be most applicable and frequently implemented in women (e.g., higher risk of iron deficiency and low bone mineral density (BMD)) and in which we have found the greatest representation of women (e.g., 112 female-only studies compared with 93 male-only studies), a lack of appreciation of the importance of sex-specific issues remains evident.

### 4.1. Calcium

Athletes who exclude dairy products or other calcium-rich foods from their diet, and/or who restrict energy intake (e.g., those experiencing disordered eating and/or low energy availability (LEA)), are at an increased risk of suboptimal calcium intake [39], which can contribute to low BMD. Among the calcium supplementation studies in this audit, 17 (34%) examined measures of bone health/BMD. However, these studies were male-dominated (1552 men vs. 329 women), and women were included in just 8/17 studies. Meanwhile, calcium studies examining other outcomes, including absorption/bioavailability, physiological adaptations, and blood pressure, were dominated by female participants (4858 women vs. 90 men).

Information on calcium dosage was available in 15/17 studies examining bone health/BMD. Consistent with recommendations for bone health (1000 mg·day^−1^ combined with 1500–2000 IU vitamin D for eumenorrheic females, 1500 mg·day^−1^ for women experiencing menstrual dysfunction or LEA [3,39]), the mean dose was 1270 ± 504 mg·day^−1^; however, none included vitamin D. Several studies investigated specific pre-exercise calcium intake (~1000 mg), providing a gut release of calcium to attenuate the bone breakdown otherwise needed to stabilise the sudden decline in serum ionic calcium at the onset of exercise [40]. Indeed, emerging confirmation that this protocol may assist bone health by reducing bone turnover, particularly in non-weight-bearing modalities [40,41], may lead to new recommendations for athletes at risk of low BMD. We note that two papers [30,42] from a single investigation of this strategy, using calcium-fortified foods to achieve the pre-exercise calcium boost, represent the only two studies of calcium supplementation in female athlete populations (Tiers 3–4) in this audit. These studies investigated gastric comfort alongside cycling performance [42], and bone turnover markers [30]. Even when the 11 studies excluded from the audit (combining calcium and vitamin D supplementation, without either in isolation) are considered, we only located 1 additional study of calcium supplementation in female athletes—a pilot investigation examining the effect of 1000 mg calcium/400 IU Vitamin D on BMD in collegiate female athletes [43]. Consequently, this audit demonstrates that despite sound hypotheses and preliminary data to support chronic (and perhaps well-timed) calcium supplementation to optimise BMD in female athletes, direct investigation and evidence of benefits are lacking.

### 4.2. Iron

Iron deficiency causes fatigue and a reduced capacity for training adaptation and recovery, alongside impaired performance [26,44]. Suboptimal iron status can result from inadequate iron intake, poor iron absorption, LEA, vegan or vegetarian diets, and increased iron losses (through menstruation, foot-strike haemolysis, sweat, and/or gastrointestinal bleeding [26,39,44]). Because women are susceptible to many of these factors, their increased requirement for iron is reflected in the current recommended intake (18 mg·day^−1^ for women and 8 mg·day^−1^ for men) [39]. Yet, iron deficiency continues to be a prevalent issue, affecting between 3–11% and 15–35% of male and female athletes, respectively [26].

In situations of iron deficiency, 100 mg·day^−1^ of elemental iron, combined with improved dietary intake, is an established treatment to increase ferritin stores [1,3,44]. A total of 54 (36%) of the audited studies examined protocols to treat iron deficiency/anaemia, with 285 females being included in 10 of the 11 studies involving athletes (Tiers 3–4). Of these 10 studies, 7 examined an oral supplementation (mean: 70 ± 58 mg·day^−1^ [12,45,46,47,48,49,50], with 4 administering <100 mg·day^−1^). Two studies investigated a 500 mg bolus dose administered intravenously [51] or intramuscularly [52], while another evaluated a dietary approach to increase iron by 15 mg·day^−1^ [53]. Participants were classified as either stage 1 or 2 iron-deficient [44] (i.e., non-anaemic) in 8 of these studies, while 2 case studies (involving 3 female athletes) investigated the treatment of stage 3 iron deficiency (anaemia) [45,46]. In summary, most investigations of the treatment of low iron status in female athletes involved oral supplements for early iron deficiency; a first line of defence using conservative (“no needles”) treatment to cover the most frequent scenarios in high-performance sport. However, further information on rapid reversal of anaemia though intravenous iron administration would be valuable to address less common, but more urgent sports-related scenarios.

Specialised interests around iron supplements in sport nutrition include strategies to optimise erythropoiesis during altitude training [54,55] and performance effects of iron supplementation. Potentially due to the niche interest, our audit located only five investigations of iron supplementation for altitude training. Here, women were well-represented (*n* = 124 women in 4 studies), with 2/4 studies involving female athletes (*n* = 96, Tier 3–4) and implementing best-practice iron treatment (105–200 mg·day^−1^ across 2–4 weeks of altitude exposure) [54,56]. Meanwhile, only 23/148 studies of iron supplementation examined performance outcomes, with just 4 involving Tier 3–4 individuals. Women were better represented with 89 participants across all four studies, while 27 men were included in just two. While all four studies examined stage 1 iron deficiency, two investigated 500 mg of intravenous [51] or intramuscular [52] elemental iron, one examined 32 mg·day^−1^ oral iron [12], and the other compared the efficacy of supplementation in elevating dietary intake [57]. Overall, important questions including whether iron deficiency without anaemia affects performance, and whether there should be sex-specific definitions of anaemia, have not been thoroughly addressed.

Optimal supplementation practices regarding iron formulation and dosing protocols remain unexplored among athletes, irrespective of sex. Of the studies audited, 29 (20%) examined different iron formulations, across a relatively equal number of men (*n* = 350) and women (*n* = 573), but no studies included athletes (Tiers 3–4). Different protocols, such as daily vs. alternate-day oral dosing, were studied in 14 investigations which were female-dominated (956 women, 27 men). However, clearly identified athletes (Tiers 3–4) were only involved in one study [56], which investigated single versus split doses of iron during altitude training among 16 female and 8 male athletes. Athletic populations should be studied independently in this context, due to the effects of exercise on iron absorption [27,28,29] making extrapolated findings from non-athletic cohorts inappropriate.

### 4.3. Vitamin D

Our primary source of vitamin D occurs from sunlight exposure [58], with athletes at risk of inadequate exposure including those with indoor or early morning/late afternoon outdoor training, athletes with dark skin pigmentation, para-athletes with missing limbs, creating less surface area for UVB absorption, and those with gastrointestinal malabsorptive conditions such as Coeliac disease [39,58]. Indeed, rates of vitamin D insufficiency as high as 84% have been reported among athletes [59,60,61]. To maintain vitamin D status, 800–2000 IU·day^−1^ is recommended between autumn and spring [3,8], while 2000–10,000 IU·day^−1^ for 1–2 months or higher weekly doses between 50,000 and 100,000 IU for 8–16 weeks might be needed to restore vitamin D status in individuals with deficiency [1,3,58]. Of the two isoforms, D3 (cholecalciferol, synthesised in human skin) supplementation is preferred over the plant-derived D2 because of its superiority in increasing and maintaining serum vitamin D concentration [62,63,64]. Where studies in our audit identified supplement formulation, most (74%) involved D3. 

We located 33 studies examining the maintenance of vitamin D status in non-deficient individuals, of which dosage was able to be determined in 31/33. Sixteen studies provided a mean daily oral dose of 3197 ± 2546 IU·day^−1^. Meanwhile, 7 studies investigated food-related vitamin D ingestion at a mean of 750 ± 275 IU·day^−1^, and 8 studies examined high-dose bolus supplementation of 44,343 ± 21,651 IU. Of the 33 studies investigating the maintenance of vitamin D status, 7 included athletes (Tiers 3–4), of which 4 included women, totalling just 72 female athletes. Studies investigating vitamin D deficiency in our audit provided treatment strategies in line with recommendations: 3242 ± 2836 IU·day^−1^ (*n* = 15) or a high bolus dose of 60,000 IU (*n* = 16). Of these 31 studies, 7 included athletes (Tiers 3–4), in which women were well-represented, being included in 6 studies (*n* = 217 women). 

Overall, guidelines for vitamin D supplementation to treat a deficiency or maintain vitamin D status in athletes are underpinned by evidence from 369 male and 289 female athletes across 14 studies. Research on this topic appears to better consider the specific needs of female athletes, without being driven by increased prevalence of insufficiency among women, as is observed for iron deficiency, and even some evidence of higher prevalence rates among men [65,66,67]. However, under-investigated themes include the effect of menstrual phase/status on Vitamin D function and bioavailability, alongside other responses to supplementation such as musculoskeletal adaptations and supplement formulations, which remain unexplored among athletes, irrespective of sex.

## 5. Conclusions

Our literature audit regarding calcium, iron, and vitamin D supplementation revealed an abundance of research among female participants, outweighing that conducted in men. The substantial representation of women in this area of sports science/medicine is welcome and provides a contrast to the under-representation in other research areas we have previously audited. To advance the field of medical supplement use by athletes, focus should be directed towards improving the quality of the literature specific to high-performance female athletes and with better methodologies to characterise menstrual cycle phase/status. Indeed, poor quality was exemplified by a universal lack of consideration for participant menstrual status combined with few studies evaluating sexual dimorphisms. While female athletes were well-represented in the evidence underpinning iron and vitamin D supplementation, there remain some unexplored nuances among athletic populations. Investigation of calcium supplementation among female athletes, however, is particularly lacking. We note that our findings do not suggest that women should not follow current sports nutrition supplementation guidelines to prevent or treat diagnosed clinical issues. Rather, new research should investigate unanswered questions around the need and response to medical supplements in both sexes, with particular attention to athletic populations alongside the methodological quality regarding menstrual status and the presence of sex differences.

## Figures and Tables

**Table 1:** Audited study supplements with method of delivery, dosage, and number of studies from which this information was determined.

Supplement	Daily Oral Supplementation		Bolus Supplementation			Food Fortification/Food Only	
	Mean Dose	No. of Studies	Mean Dose	Dosing Method	No. of Studies	Mean Dose	No. of Studies
**Calcium**	1264 mg	16	-	-	-	798 mg	9
**Iron**	100 mg	100	846 mg	IV (*n* = 16) IM (*n* = 3)	19	9.2 mg	23
**Vitamin D**	2754 IU	54	83,709 IU	Oral (*n* = 38) IV (*n* = 2) IM (*n* = 2)	42	731 IU	8

Note, the total number of studies does not equal the total number of studies audited, as it was not possible to determine supplement dose in all studies. There were no studies investigating a bolus calcium supplementation regime as such; rather, studies administering a one-off calcium dose aimed to evaluate acute pre-exercise supplementation or a one-off calcium infusion. Dosage refers to that of elemental calcium and iron. IV = intravenous, IM = intramuscular.

## Data Availability

Data (metrics extracted for analysis across the 299 studies) are available from the corresponding author (ella.smith@acu.edu.au) upon reasonable request. Reuse is permitted with appropriate reference to this paper.

## Supplementary Materials

The following supporting information can be downloaded at: https://www.mdpi.com/article/10.3390/nu14163372/s1, Figure S1: Calcium flowchart. Male (M), female (F), menstruating (men), hormonal contraception (HC). Bold text denotes papers, non-bold typeface is used for studies. From section B onwards, all counts, and percentages refer to individual studies (not papers). The methodological standard of classified studies in metric C, step 2 excludes pregnant women. * studies comparing unclassified vs. Tier 1, ^ studies comparing unclassified vs. Tier 2, ◊ studies comparing tier 0 vs. 2. Figure S2: Iron flowchart. Male (M), female (F), menstruating (men), hormonal contraception (HC). Bold text denotes papers, non-bold typeface is used for studies. From section B onwards, all counts, and percentages refer to individual studies (not papers). The methodological standard of classified studies in metric C, step 2 excludes pregnant women. † studies comparing unclassified vs. tier 4. Figure S3: Vitamin D flowchart. Male (M), female (F), menstruating (men), hormonal contraception (HC). Bold text denotes papers, non-bold typeface is used for studies. From section B onwards, all counts, and percentages refer to individual studies (not papers). The methodological standard of classified studies in metric C, step 2 excludes pregnant women.
