# Supplementary figures and images for: Managing Female Athlete Health: Auditing the Representation of Female versus Male Participants among Research in Supplements to Manage Diagnosed Micronutrient Issues
Source: Nutrients. 2022 Aug 17;14(16):3372. doi: 10.3390/nu14163372 (PMC9412577; doi:10.3390/nu14163372)

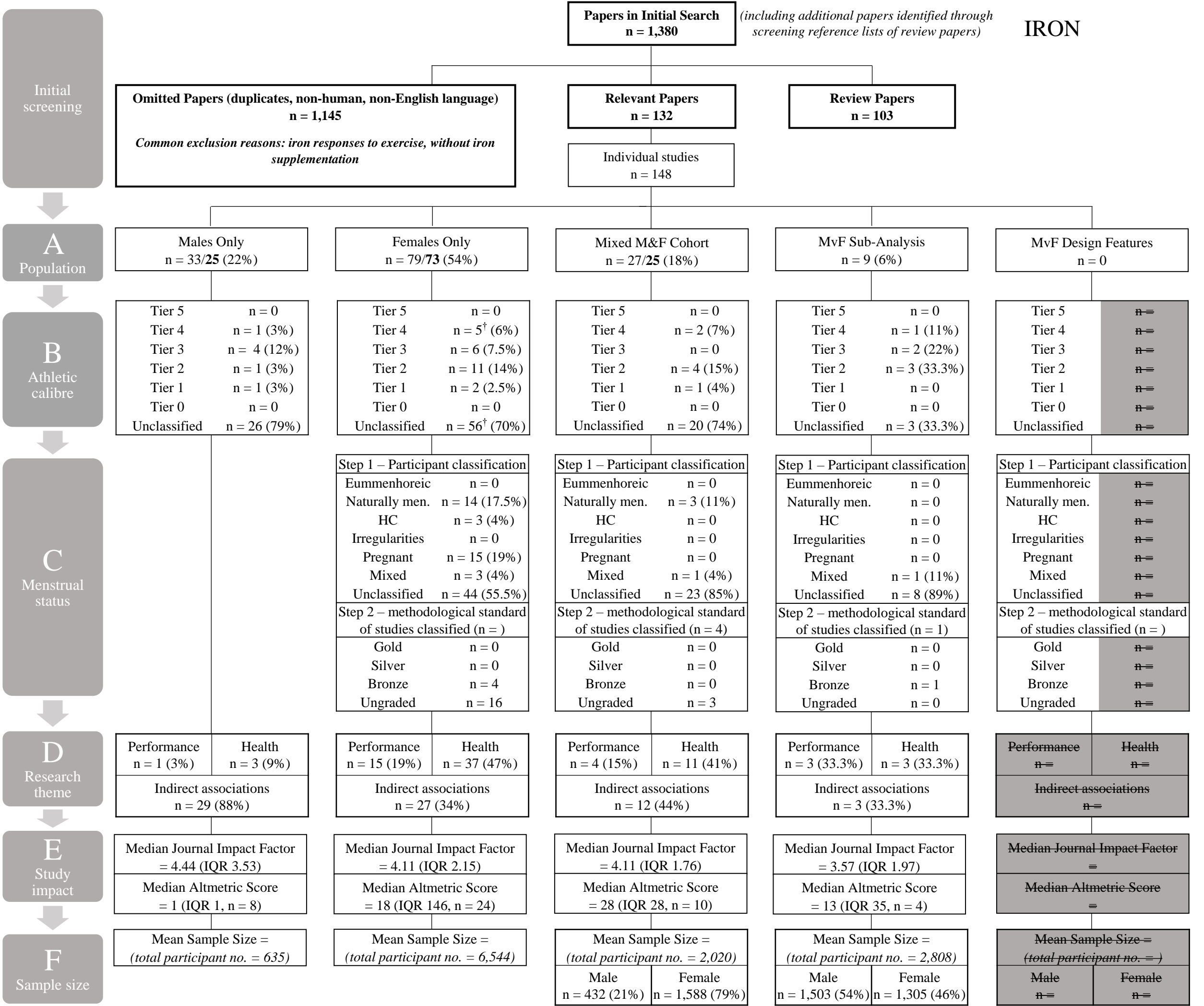

Supplement: Supplementary file 1 [file nutrients-14-03372-s001.zip › nutrients-1858959-supplementary.pdf]
